# The population-specific Thr44Met OCT3 coding variant affects metformin pharmacokinetics with subsequent effects on insulin sensitivity in C57Bl/6J mice

**DOI:** 10.1007/s00125-024-06287-1

**Published:** 2024-10-18

**Authors:** Qian Wang, Megan P. Leask, Kate Lee, Jagdish Jaiswal, Prasanna Kallingappa, Waruni Dissanayake, Chris Puli’uvea, Conor O’Sullivan, Huti Watson, Phillip Wilcox, Rinki Murphy, Troy L. Merry, Peter R. Shepherd

**Affiliations:** 1 Department of Molecular Medicine and Pathology, University of Auckland, Auckland, New Zealand; 2 Maurice Wilkins Centre, Auckland, New Zealand; 3 University of Alabama, Birmingham, AL USA; 4 Auckland Cancer Society Research Centre, University of Auckland, Auckland, New Zealand; 5 Department of Biomedicine and Diagnostics, Auckland University of Technology, Auckland, New Zealand; 6 Moko Foundation, Kaitaia, New Zealand; 7 Paratene Ngata Research Centre, Ngati Porou Oranga, Te Puia Springs, New Zealand; 8 Department of Statistics, University of Otago, Dunedin, New Zealand; 9 Department of Medicine, University of Auckland, Auckland, New Zealand; 10 Auckland Diabetes Centre, Te Whatu Ora Health New Zealand, Te Toka Tumai, New Zealand; 11 Department of Nutrition, University of Auckland, Auckland, New Zealand

**Keywords:** Diabetes, GDF-15, Insulin sensitivity, Metformin, Pharmacogenetics, Precision medicine, *SLC22A3*

## Abstract

**Aims/hypothesis:**

Metformin is an important first-line treatment for type 2 diabetes and acts by increasing the body’s ability to dispose of glucose. Metformin’s efficacy can be affected by genetic variants in the transporters that regulate its uptake into cells. The *SLC22A3* gene (also known as *EMT; EMTH; OCT3*) codes for organic cation transporter 3 (OCT3), which is a broad-specificity cation transporter that also transports metformin. Most *SLC22A3* variants reduce the rate of metformin transport but the rs8187715 variant (p.Thr44Met) is reported to increase uptake of metformin in vitro. However, the impact of this on in vivo metformin transport and efficacy is unknown. Very few carriers of this variant have been reported globally, but, notably, all were of Pacific Island descent. Therefore, this study aims to understand the prevalence of this variant in Polynesian peoples (Māori and Pacific peoples) and to understand its impact on metformin transport and efficacy in vivo.

**Methods:**

rs8187715 was genotyped in 310 individuals with Māori and Pacific ancestry recruited in Aotearoa New Zealand. To study this variant in a physiological context, an orthologous knockin mouse model with C57BL/6J background was used. Pharmacokinetic analysis compared uptake rate of metformin into tissues. Plasma growth/differentiation factor 15 (GDF-15) was also measured as a marker of metformin efficacy. Glucose and insulin tolerance was assessed after acute or sustained metformin treatment in knockin and wild-type control mice to examine the impact of the variant on metformin’s glycaemic control.

**Results:**

The minor allele frequency of this variant in the Māori and Pacific participants was 15.4%. There was no association of the variant with common metabolic parameters including diabetes status, BMI, blood pressure, lipids, or blood glucose and HbA_1c_. However, in the orthologous knockin mouse model, the rate of metformin uptake into the blood and tissues was increased. Acute metformin dosing increased insulin sensitivity in variant knockin mice but this effect was lost after longer-term metformin treatment. Metformin’s effects on GDF-15 levels were also lost in variant knockin mice with longer-term metformin treatment.

**Conclusions/interpretation:**

These data provide evidence that the *SLC22A3* rs8187715 variant accelerates metformin uptake rate in vivo. While this acutely improves insulin sensitivity, there was no increased effect of metformin with longer-term dosing. Thus, our finding of a high prevalence of this variant specifically in Māori and Pacific peoples identifies it as a potential population-specific pharmacogenetic marker with potential to guide metformin therapy in these peoples.

**Graphical Abstract:**

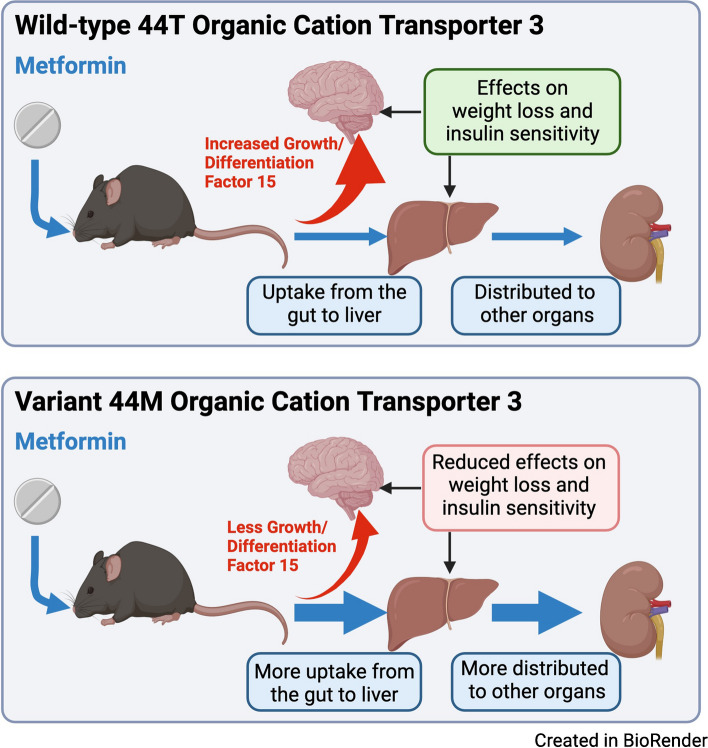

**Supplementary Information:**

The online version contains peer-reviewed but unedited supplementary material available at 10.1007/s00125-024-06287-1.


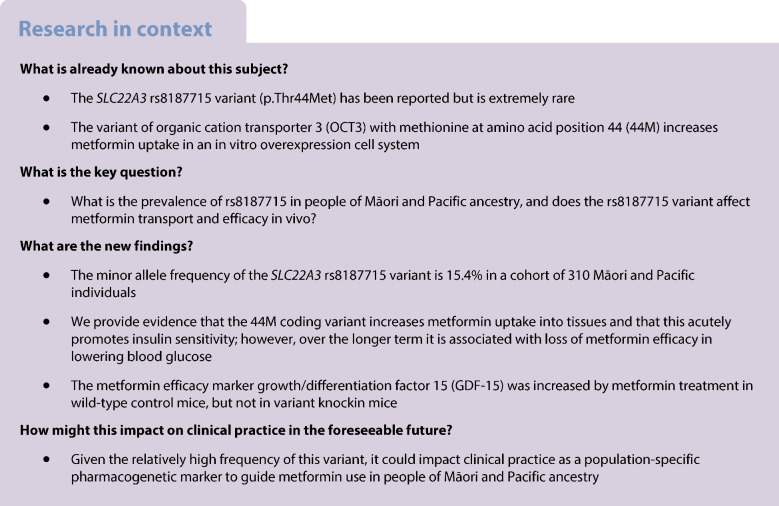


## Introduction

Type 2 diabetes is a severe and growing public health concern that impacted approximately 462 million individuals in 2017 [1]. To date, the treatments for this disease have generally used algorithmic ‘one size fits all’ strategies, but there are growing efforts globally to develop precision medicine approaches to better meet the needs of individual patients [2]. However, these studies have been largely undertaken in the USA and Europe and so results may not be fully translatable to genetically diverse ethnic populations across the planet [3]. One such population is the Polynesian peoples of the Pacific (including New Zealand [NZ] Māori), where a range of gene coding variants have been identified that have definable physiological impacts and are common in these peoples but virtually absent in other populations worldwide [4–6].

Metformin is the predominant first-line treatment for patients with type 2 diabetes, as it produces a durable anti-hyperglycaemic effect and beneficial effects on lipids and blood pressure, and small but consistent weight loss [7]. Multiple studies have shown that metformin regulates blood glucose levels by reducing hepatic gluconeogenesis and acts in muscle to improve the efficacy of insulin [8–12]. The underlying mechanisms of metformin efficacy, in part, involve impacts on insulin signalling and mitochondrial function in insulin target tissues [8–12]. However, other mechanisms have also been implicated. Accumulating data suggest that metformin also modulates glucagon-like peptide 1 (GLP-1) and microbiota composition in the gut system [13, 14]. The metformin effects on weight [7] involve stimulation of growth/differentiation factor 15 (GDF-15) production in the gut and a consequent anorectic effect mediated via the hindbrain [15–17]. Genetically driven differences in responses to metformin is an area identified as a potential marker for precision medicine strategies [2].

Under physiological conditions metformin has a positive charge, making it difficult for metformin to pass through cellular membranes; thus, transporters are required. Organic cation transporters (OCTs), multidrug and toxin extruders (MATEs) and plasma membrane monoamine transporter (PMAT) have been reported to be involved in the transport of metformin across biological membranes. Organic cation transporter 3 (OCT3) (encoded by gene *SLC22A3*; also known as *EMT; EMTH; OCT3*) plays an important role in this process. After oral dosing, metformin is absorbed into gut cells through PMAT (encoded by gene *SLC29A4*) and OCT3 which are expressed in the brush border of the enterocytes [18, 19]. In liver, organic cation transporter 1 (OCT1) and OCT3 are responsible for the hepatic uptake of metformin, and these are expressed on the basolateral membrane of hepatocytes [20–22]. Recent studies have identified genetic variants that affect metformin efficacy and this has generated much interest in the potential for such genotypes to be used in guiding precision medicine strategies for treating type 2 diabetes [2, 23].

OCT3 is a broad-specificity cation transporter [24] and polymorphisms in the *SLC22A3* gene have been found to influence metformin pharmacokinetics, which, in turn, affect patient response to the drug [25–28]. A study of metformin efficacy in the USA identified a small number of participants with the *SLC22A3* rs8187715 variant that causes a p.Thr44Met coding change [26]. Notably, all those who were carriers were reported to be from the Pacific Islands and the variant was not present in other participants and is very rare in global genomic databases [26, 29]. These researchers found that the OCT3 p.Thr44Met variant increased the uptake of metformin in an in vitro cell-based overexpression system [26]. This is the first *SLC22A3* variant found to increase metformin transport and the effects of an increase in metformin transport in vivo are unknown.

Here we study the incidence of the rs8187715 variant in Māori and other Polynesian peoples. We went on to examine the impact of the variant on metformin efficacy in vivo by using an orthologous knockin (KI) mouse model.

## Methods

### Study cohort, genotyping and association analyses

Genotype frequency and associations with metabolic parameters were derived from the Māori and Pacific peoples’ deep phenotyping cohort that we have described previously [5]. The study recruited young, healthy self-reported male volunteers with one or more NZ Māori or Polynesian grandparents at four sites in NZ. These included the Universities of Auckland and Otago. The study also included two Māori-led organisations in regional areas of the country, the Moko Foundation and Ngati Porou Oranga, a Māori Primary Health Organisation (PHO) delivering primary healthcare across Ngati Porou, who identified diabetes and its treatment as a priority area for research. All participants provided written informed consent for the collection of samples and subsequent analysis. Handling and disposing of human samples at all sites were guided by Māori tikanga protocols provided by Māori research partner organisations. Genotyping was carried out using the Sequenom MassArray (Agena Bioscience, San Diego, USA) as previously described [5]. All association analyses described below were carried out using the RStudio (RStudio v4.0.2 statistical software, www.rstudio.com) as previously described, with analyses adjusted for age and ethnicity as assessed by grandparent ancestry as we have previously described [5]. Ethical approval was given by the Health and Disability Ethics Committee (17STH79) [5].

### Animals

All animal experimental protocols were approved by the University of Auckland Animal Ethics Committee, were performed in accordance with the NZ Animal Welfare Act (1999) and the Guide for the Care and Use of Laboratory Animals, and complied with the ARRIVE guidelines. Mice were maintained at temperatures of 22 ± 2°C on a 12 h light/dark schedule with a 30 min dawn/dusk phase. Mice were group-housed with ad libitum access to food and water unless otherwise indicated. The chow diet was Teklad 2018 (Inotiv, USA), 18% protein rodent diet.

### Generation of KI mice

**Fig. 1:**
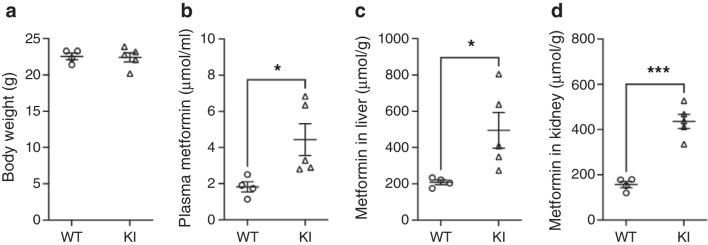
The *SLC22A3* variant increased metformin uptake rate after oral administration. Mice of similar body weight (**a**) were administered with a single dose of metformin (15 mg/kg) via oral gavage (WT *n*=4, KI *n*=5). Metformin contents in plasma (**b**), liver (**c**) and kidney (**d**) were measured via LC-MS/MS. All error bars are SEM. All statistics are by two-tailed unpaired *t* test. *p<0.05 and ***p<0.001

Protein sequence alignment of OCT3 shows the amino acid at 44 position is serine (Ser) in most mammalian species, while in humans it is threonine (Thr) (electronic supplementary material [ESM] Fig. 1). However, these two amino acid residues are considered conservative substitutions, suggesting both are functionally important and that the phenotype could be replicated in a mouse model. The Ser44Met mice on the C57BL/6J background were generated by the Rodent Genome Engineering Resource (ROGER) Facility (Faculty of Medical and Health Sciences, University of Auckland, NZ) using CRISPR/Cas9 technology, and the targeting strategy is shown in ESM Fig. 1. To introduce the serine-to-methionine mutation, single guide RNA (sgRNA) and donor sequences were selected and co-injected into C57BL/6J embryos (ESM Fig. 1b). The proper insertion of the *Slc22a3* c.131–132 GC-to-TG mutation was confirmed by DNA Sanger sequencing of the founder mice (ESM Fig. 1c). Sequencing was used to verify the presence of the variant and lack of non-intended changes in the surrounding region (Forward 5′-ATGCCCACGTTCGACCA-3′; Reverse 5′-GTTCAGGATGGCTTGGGTGA-3′). CRISPOR online software (accessed in January 2019) [30] was used to check the specificity of the guide RNA sequences. Mouse genotyping was performed by either: (1) Sanger sequencing (Massey Genome Service, NZ); or (2) commercial genotyping using real-time PCR (Transnetyx, TN, USA). The mouse colony was backcrossed five times with wild-type (WT) C57BL/6J mice to remove unwanted mutations that may have arisen due to the gene editing process. Animals used in the experiments were all from homozygous breeders. Each pair of breeders was only used to breed a maximum of three litters. Mice were randomised to experimental conditions for all experiments.

### Metformin pharmacokinetics study

Metformin pharmacokinetics were tested by using two different dosages: low dose (15 mg/kg) and high dose (200 mg/kg). For low dose, 7–8-week-old male mice and 20–25-week-old female mice of both genotypes (WT and KI) were used. For high dose, 20–25-week-old male mice of both genotypes were used. All the animals were fasted for 6 h from 06:00 hours. Then, mice were administered a single dose of metformin (15 mg/kg or 200 mg/kg) via oral gavage. Blood samples were collected by cardiac puncture 30 min after metformin administration and centrifuged at 2000 rev/min for 15 min at 4°C. Plasma was collected and stored at −80°C until further analysis.

### Metformin tissue content measurement through LC-MS/MS

First, 50 mg of liver or kidney tissue was homogenised with 5 volumes of Milli-Q water by TissueLyser (QIAGEN, the Netherlands). Then, 10 µl of tissue homogenate or plasma was mixed with 40 µl of ice-cold solution of methanol:acetonitrile (1:1 v/v) containing atenolol (0.25 µmol/l) and centrifuged at 12,000 rev/min for 5 min. Next, 40 µl of the supernatant was mixed with 40 µl of ammonium formate (7 mmol/l pH 3.5) and loaded into HPLC vials, and 10 µl was injected into the LC-MS/MS system. An Agilent 1200* series HPLC (Agilent, USA) system equipped with a Synergy Polar-RP 100 Å column (100 mm × 3 mm, Phenomenex, USA) was used for chromatographic separation of metformin and atenolol (internal standard). The mobile phase consisted of 7 mmol/l ammonium formate containing 0.1% formic acid in Milli-Q water and 7 mmol/l ammonium formate containing 0.1% formic acid in 80% acetonitrile. The mobile phase flow rate was set to 0.6 ml/min. Gradient elution was used for 5 min, and the flow rate was maintained at 0.6 ml/min throughout the gradient. Column oven temperature and autosampler temperature were set to 35°C and 4°C, respectively. Test samples outside the calibration range were diluted with either blank plasma or blank matched tissue homogenate. Metformin concentration in mouse plasma and tissues was measured by LC-MS/MS using a 6460 triple quadrupole mass spectrometer (Agilent, Santa Clara, USA) equipped with a Jet Stream electrospray source (Agilent, USA) operated in positive ion mode.

### Single-dose metformin oral GTT and IPITT

Adult (20–25 weeks) mice of both sexes and genotypes were randomly assigned for the experiment. Mice were fasted for 6 h (07:30 hours to 13:30 hours) in a clean cage with wood chip bedding and free access to water. Blood glucose levels were measured through tail vain blood sampling. Blood samples of 20 µl were taken before oral GTT as time 0. A single dose of metformin (200 mg/kg) was given to the mice via oral gavage. Then, a single dose of glucose (2 g/kg) was given within 20 min after metformin administration. Blood glucose levels were then checked at 15, 30, 60, 90 and 120 min. Blood samples of 20 µl were collected at 30, 60 and 90 min for plasma insulin level measurement. Blood samples were centrifuged at 2000 rev/min for 15 min at 4°C and plasma samples were stored at −80°C until used for insulin assay, following the manufacturer’s instructions. A mouse insulin ELISA kit was obtained from Mercodia (cat. 10-1247-01, Sweden). After a week’s wash out time, the same groups of mice were fasted for 4 h (07:30 hours to 11:30 hours) in a clean cage with wood chip bedding. Baseline blood glucose was measured. Then, mice were given an i.p. injection of insulin (0.5 U/kg, Actrapid, NovoNordisk, Denmark) 20 min after a dose of metformin (200 mg/kg). Serial mouse glucose levels were measured at the same time points as indicated above. The blood glucose concentration was plotted against time and the AUC was calculated. AUC above baseline blood glucose was calculated for GTT, while AUC below baseline blood glucose was calculated for ITT.

### Longer-term metformin treatment study

Adult mice (20–25 weeks) of both sexes and genotypes were administered with 200 mg/kg metformin daily at 07:00 hours. At 1 day before the start of metformin administration, blood samples were collected through the tail vein as a basal control. Body weight was examined daily, and blood glucose was checked every 2 days in the morning before metformin oral gavage using a glucometer and glucose strips (Accu-Check, Roche, Switzerland). GTTs were performed on day 10 of metformin treatment. ITTs were carried out on day 14 of the experiment. Blood samples were collected at the end of the experiment by cardiac puncture and centrifuged at 2000 rev/min for 15 min at 4°C. GDF-15 levels were measured using a mouse GDF-15 ELISA (R&D Systems, USA) following the manufacturer’s instructions.

### High-fat diet study

Mice 8–9 weeks old of both sexes and both genotypes were fed with a high-fat diet (HFD) (60% fat by energy content; SF13-092, Specialty Feeds, Australia) for 17 weeks. Mice were randomised into two groups per sex and genotype to receive either 200 mg/kg metformin or water daily via oral gavage. At the end of the experiment, blood glucose was measured after 6 h of fasting. Blood samples were collected at the end of the experiment by cardiac puncture and centrifuged at 2000 rev/min for 15 min at 4°C. GDF-15 levels were measured using a mouse GDF-15 ELISA (R&D, MGD150) following the manufacturer’s instructions.

### Statistical analyses

Statistical analyses were performed using Prism 9 (Dotmatics, USA), using unpaired two-tailed *t* tests, or two-way ANOVA with Sidak’s or Tukey’s multiple comparisons test. All error bars are SEM. All statistics show *p<0.05, **p<0.01 and ***p<0.001.

### Oversight of findings

Given that this study involves a population-specific gene variant, Māori and Pacific researchers from the Universities of Auckland and Otago (MPL, CP, PW) along with staff from the Moko Foundation (CO’S) and Ngati Porou Oranga (HW) were involved in reviewing conclusions drawn from the data to ensure the findings of the study did not portray harmful narratives.

## Results

### rs8187715 *SLC22A3* coding variant is relatively common in Māori and other Polynesian populations of the Pacific

The minor allele (T) frequency of rs8187715 is reported as <0.01 in the Genome Aggregation Database (gnomAD) [29] (v.2.1.1). Specifically, only one T allele was identified in an individual of East Asian ancestry out of 5761 individuals of East Asian ancestry. Genotyping in a cohort of 310 individuals of Māori and Pacific ancestry found that the minor allele frequency was 15.4% (Table 1). This proportion was similar in the NZ Māori subgroup (*n*=123, 17.0%) and the subgroup made up of Polynesian peoples from other Pacific Islands (*n*=187, 14.4%). Since this cohort was mainly people under the age of 40 and without disease, information on metformin efficacy was not available. However, there was no association with fasting levels of glucose, insulin, lipids, blood pressure or HbA_1c_, suggesting the variant was not a driver of major impairments in glucose metabolism (Table 1). This remained the case when adjustments were made for age, BMI and ancestry (Table 2).

**Table 1:** Characteristics of the study population by genotypes

Characteristic	CC	CA/AA
*n*	263	48
Age (yr)	26.7 ± 6.5	28.5 ± 6.3
Weight (kg)	104.6 ± 19.8	106.6 ± 19.2
Height (cm)	181.3 ± 6.1	180.7 ± 7.1
BMI (kg/m^2^)	31.7 ± 5.4	32.5 ± 5.4
Glucose (mmol/l)	5.7 ± 0.8	5.5 ± 0.9
Insulin (pmol/l)	101.4 ± 187.8	85.8 ± 78.6
HbA_1c_ (mmol/mol)	35.2 ± 3.4	35.7 ± 4.6
HbA_1c_ (%)	5.4 ± 0.5	5.4 ± 0.5
Cholesterol (mmol/l)	4.7 ± 1.1	4.9 ± 1.1
LDL (mmol/l)	3.1 ± 1.0	3.2 ± 1.0
HDL (mmol/l)	1.2 ± 0.3	1.2 ± 0.3
BP (systolic mm/Hg)	129.9 ± 13.4	129.5 ± 12.1
BP (diastolic mm/Hg)	78.2 ± 10.3	78.9 ± 8.8

Data are shown as mean ± SD

**Table 2:** Association of rs8187715 with HbA_1c_ and fasting levels of glucose and insulin, with adjustments including ethnicity

	Variant* n* (CC/CA/AA)	Age, ETH adjusted			BMI, age, ETH adjusted		
		β	SE	*p* value	β	SE	*p* value
Glucose (mmol/l)	256/38/9	0.05	0.08	0.594	0.04	0.08	0.671
Insulin (pmol/l)	245/38/9	−1.43	2.97	0.630	−1.70	2.94	0.563
HbA_1c_ (mmol/mol)	259/36/9	−0.18	0.45	0.688	−0.23	0.44	0.577

The association of rs8187715 SNP with metabolic parameters was assessed in a previously described cohort of Māori and Pacific male participants [5]

ETH, ethnicity

### Increased metformin distribution in *Slc22a3* KI mice

In body weight-matched mice, metformin plasma content was increased in KI mice compared with that of WT mice 30 min after oral gavage of 15 mg/kg metformin (Fig. 1a, b) (mean ± SEM WT: 1.8 ± 0.3 μmol/ml; KI: 4.4 ± 0.9 μmol/ml; *p*=0.04). We also examined metformin content in the liver and kidney 30 min after this dose. As shown in Fig. 1c, d, both liver and kidney metformin content were higher in KI mice compared with those in WT mice (mean ± SEM WT liver vs KI liver: 208.7 ± 12.8 µmol/g vs 494.8 ± 98.6 μmol/g; *p*=0.04; WT kidney vs KI kidney: 156.9 ± 14.0 μmol/g vs 436.2 ± 31.7 μmol/g; *p*=0.0003). A similar pattern of increased metformin uptake was observed in female mice (ESM Fig. 2) in liver and kidney (ESM Fig. 2c, d). Studies examining the biological function of metformin in mice often use a higher dose of metformin, typically 200 mg/kg. An oral gavage of this dose of metformin also produced a consistent pattern of increased metformin uptake in plasma, liver and kidney in the KI mice (ESM Fig. 3).

### Increased insulin sensitivity in single-dose metformin-treated KI mice

**Fig. 2:**
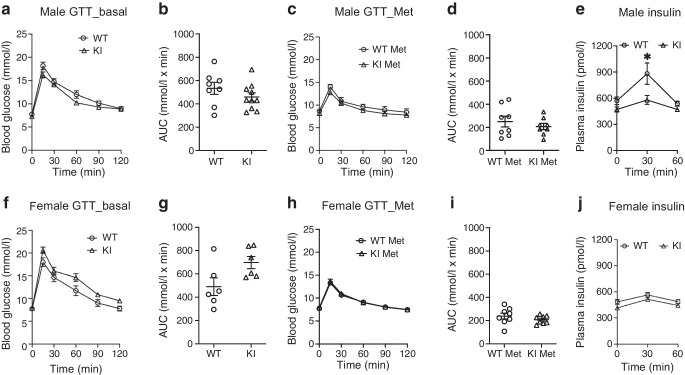
Acute dose of metformin increased insulin sensitivity in GTTs in variant KI mice. GTTs of both male (**a**) and female (**f**) mice without metformin treatment. The glucose AUC of each individual mouse was calculated (**b** and **g**). Male WT *n*=8; male KI *n*=10; female WT *n*=6; female KI *n*=6. GTTs of both male (**c**) and female (**h**) mice after a single acute dose of metformin (200 mg/kg). The glucose AUC of each individual mouse was calculated (**d** and **i**). Plasma insulin levels of both male (**e**) and female (**j**) mice. Male WT *n*=8; male KI *n*=8; female WT *n*=8; female KI *n*=8. All error bars are SEM. Statistics of AUC are by two-tailed unpaired *t* test with Welch’s correction. Statistics of blood glucose and plasma insulin levels are by two-way ANOVA with Sidak’s multiple comparisons test. *p<0.05. Met, metformin

**Fig. 3:**
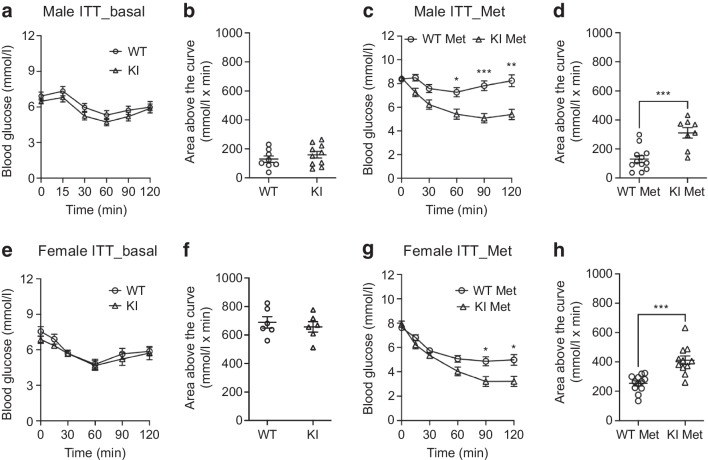
Acute dose of metformin increased insulin sensitivity in ITTs in variant KI mice. ITTs of both male (**a**) and female (**e**) mice without metformin treatment. The glucose area above the curve of each individual mouse was calculated (**b** and **f**). Male WT *n*=8; male KI *n*=10; female WT *n*=6; female KI *n*=6. ITTs of both male (**c**) and female (**g**) mice after a single acute dose of metformin (200 mg/kg). The glucose area above the curve of each individual mouse was calculated (**d** and **h**). Male WT *n*=12; male KI *n*=8; female WT *n*=12; female KI *n*=11. All error bars are SEM. Statistics of area above the curve are by two-tailed unpaired *t* test with Welch’s correction. Statistics of blood glucose levels are by two-way ANOVA with Sidak’s multiple comparisons test. *p<0.05, **p<0.01 and ***p<0.001. Met, metformin

We next assessed the basal blood glucose levels and insulin sensitivity by GTT and ITT. Glucose and insulin tolerance were similar in untreated WT and KI mice, showing the variant per se did not affect glucose metabolism (Fig. 2a, b), which is consistent with the human data. Following a single dose of 200 mg/kg metformin, there was no difference in blood glucose levels between WT and KI mice in a GTT (Fig. 2c, d). However, in this GTT, plasma insulin levels were increased in WT male mice but not in KI mice at 30 min (Fig. 2e) (mean ± SEM WT: 881.5 ± 122.5 pmol/l; KI: 580.6 ± 51.2 pmol/l; *p*=0.05). The pattern was similar in female mice (Fig. 2f–j). This suggested the KI animals were more insulin sensitive and to test this we performed ITTs. In untreated WT and KI mice, insulin tolerance was similar, showing the variant per se did not affect insulin sensitivity (Fig. 3a, b, e, f). However, we found blood glucose levels were lower in metformin-treated KI mice than in WT mice of both sexes, confirming increased insulin sensitivity (Fig. 3c, d, g, h) (area above the curve [mmol/l × min]: mean ± SEM male WT vs KI: 130.6 ± 23.5 vs 311.1 ± 26.4; *p*=0.0006; female WT vs KI: 254.0 ± 16.5 vs 411.3 ± 29.6; *p*=0.0003).

### Metformin efficacy was attenuated in KI mice after sustained metformin treatment

**Fig. 4:**
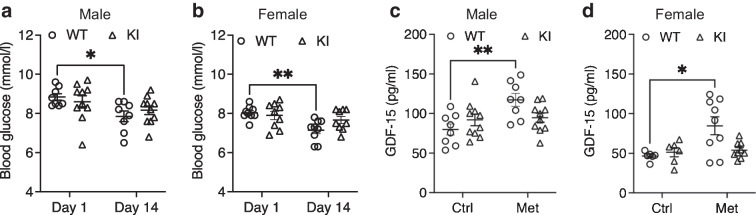
Longer-term metformin treatment was less effective in decreasing blood glucose and GDF-15 levels in KI mice. We measured blood glucose levels in male (**a**) and female (**b**) mice before (day 1) and after 2 weeks (day 14) of metformin treatment. Male WT *n*=8; male KI *n*=10; female WT *n*=9; female KI *n*=9. GDF-15 levels were measured in control groups at baseline and in 2 weeks metformin treated groups in male (**c**) and female (**d**) mice. Male Ctrl group: WT *n*=8; KI *n*=10; female Ctrl group: WT *n*=6; KI *n*=6. All error bars are SEM. Statistics of blood glucose and GDF-15 levels are by two-way ANOVA with Sidak’s multiple comparisons test. *p<0.05 and **p<0.01. Ctrl, control; Met, metformin

While the acute treatment effects suggested that metformin might be more efficacious in the short term, this needed to be tested with longer-term dosing studies. Following 14 days of metformin treatment, both male and female WT mice showed reduced non-fasted blood glucose levels as expected (mean ± SEM day 1 vs day 14 [mmol/l]: male 8.8 ± 0.2 vs 7.9 ± 0.3; *p*=0.02; female 8.0 ± 0.1 vs 7.1 ± 0.2; *p*=0.004). However, this was not seen in the KI mice post treatment (Fig. 4a, b). To understand whether this correlated with another biomarker of metformin action, we measured plasma GDF-15 levels at baseline and after 2 weeks of metformin treatment. Plasma GDF-15 was increased in WT mice after 14 days of metformin treatment (mean ± SEM control vs metformin [pg/ml]: male 79.9 ± 7.0 vs 117.3 ± 8.2; *p*=0.002; female 46.6 ± 2.323 vs 84.7 ± 11.2; *p*=0.01), but there was no difference in either male or female KI mice (Fig. 4c, d). Plasma lactate levels were not changed after 14 days of metformin treatment (ESM Fig. 4).

**Fig. 5:**
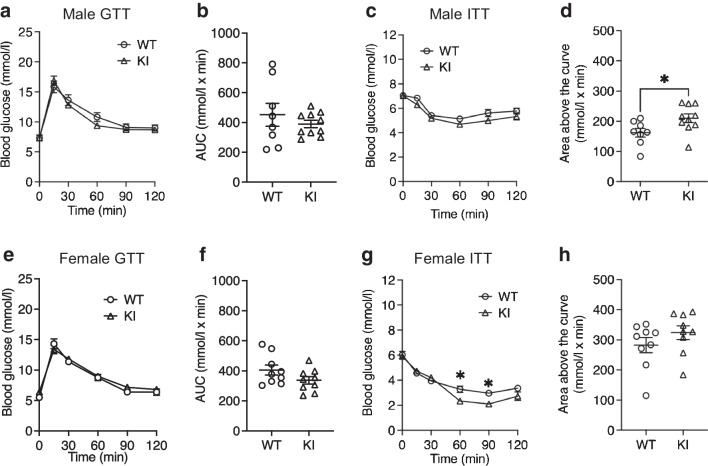
Longer-term metformin treatment was less effective in increasing insulin sensitivity. GTTs and ITTs were checked in both male (**a**–**d**; WT *n*=8, KI *n*=10) and female (**e**–**h**; WT *n*=9; KI *n*=9) mice after 2 weeks of metformin treatment. All error bars are SEM. Statistics of AUC are by two-tailed unpaired *t* test with Welch’s correction. Statistics of blood glucose levels are by two-way ANOVA with Sidak’s multiple comparisons test. *p<0.05

There was no difference between KI and WT mice in glucose tolerance after this longer-term metformin treatment (Fig. 5a, b, e, f). While a single acute dose of metformin had increased insulin tolerance in KI mice relative to WT mice (Fig. 3a, b, e, f), after 2 weeks of metformin treatment we found that this effect was lost (Fig. 5c, d, g, h).

**Fig. 6:**
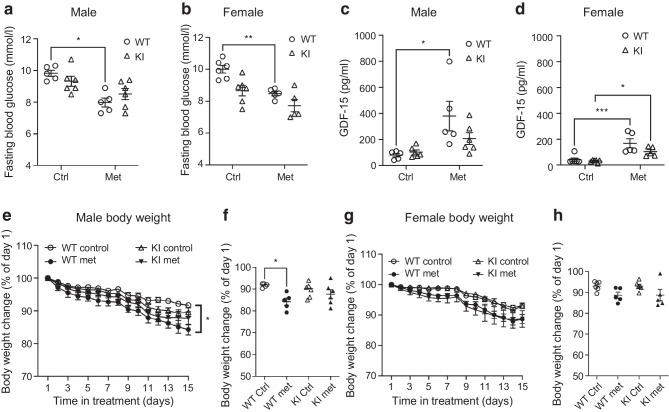
Longer-term metformin treatment was less effective in increasing plasma GDF-15 and decreasing blood glucose in KI mice fed an HFD. Blood glucose (**a**, **b**), GDF-15 levels (**c**, **d**) and weight (**e**–**h**) were assessed in both HFD-fed male (**a**, **c**, **e**, **f**) and female (**b**, **d**, **g**, **h**) mice with 2 weeks of metformin or water treatment via oral gavage. Male WT: both Ctrl and Met groups *n*=5; male KI: both Ctrl and Met groups *n*=6; female Ctrl group: both WT and KI *n*=6; female Met group: both WT and KI *n*=5. All error bars are SEM. Statistical analysis used two-way ANOVA with Sidak’s multiple comparisons test. *p<0.05, **p<0.01 and ***p<0.001. Ctrl, control; Met, metformin

Metformin is most usually used in the contexts of obesity and insulin resistance and has slightly different kinetics and impact in individuals with and without diabetes. Therefore, we replicated experiments in mice fed an HFD to induce obesity (although not a diabetic state per se). Plasma blood glucose was decreased in WT obese mice after 2 weeks of metformin treatment compared with control groups in both males and females (mean ± SEM control vs metformin [mmol/l]: WT male 9.8 ± 0.2 vs 8.0 ± 0.3; *p*=0.02; WT female 10.0 ± 0.2 vs 8.5 ± 0.1; *p*=0.003), but, as with the chow-fed mice, there was no reduction in the KI mice (Fig. 6a, b). A similar pattern compared with chow diet was also seen in GDF-15 levels, with these increasing in WT mice (control vs metformin [pg/ml]: male 79.9 ± 13.8 vs 380.0 ± 113.5; *p*=0.01; female 42.2 ± 12.9 vs 168.5 ± 35.7; *p*=0.0004) but to a much lesser extent in KI mice (Fig. 6c, d). Metformin induced a reduction in weight in male control WT mice fed an HFD (mean ± SEM control vs metformin [% of body weight on day 1]: 91.7 ± 0.4% vs 84.3 ± 1.6%; *p*=0.02) but this was not seen in KI mice (Fig. 6e–h).

## Discussion

The most important finding of the current study is that the variant with methionine at amino acid position 44 (44M) of OCT3 can alter the disposition of metformin and its efficacy in vivo. We find in the mice homozygous for the 44M version of OCT3 that metformin uptake is increased into target tissues and that insulin sensitivity is improved after an acute dose. This is consistent with the large body of data showing that metformin increases insulin sensitivity [31–34]. Surprisingly, although this improvement in metformin efficacy is seen with a single acute dose, it is lost after longer-term metformin treatment in mice fed with both a chow diet and an HFD to induce an obese state. This was observed in the impacts of metformin on both insulin sensitivity and GDF-15 levels, and in the weight reductions resulting from metformin treatment. This suggests that impacts of metformin on both peripheral glucose metabolism and the gut are lost in the OCT3 44M mice. This could be due to the variant increasing metformin turnover, which would result in an impairment of metformin effects on glucose metabolism over the longer term.

Another important finding is the sex-specific differences. In our studies the effects of the OCT3 44M version are stronger in male mice than females. The reasons for this are not clear but sex differences in glucose metabolism have been observed in both rodents and humans [35, 36], suggesting that sex is an important factor, affecting sex differences in animals and humans. In this regard it is worth noting that oestradiol is known to inhibit the monoamine transport activity of OCT3 [37].

A major strength of our study is that it provides evidence to establish the in vivo relevance of the OCT3 p.Thr44Met variant at physiological tissue expression levels in both male and female animals. A major weakness is that this has yet to be shown in humans. Genetic variations related to metformin pharmacokinetics and pharmacodynamics are known to be associated with the substantial inter-individual variation in glucose lowering efficacy of metformin treatment [38]. Several studies have discovered polymorphisms in the *SLC22A3* gene that affect the metformin transport of OCT3. However, most of these variants are reported to reduce metformin transport efficiency [26, 28, 39–43]. Among these, four common polymorphisms (rs2292334, rs3088442, rs555754 and rs376563) have been found in European, African, Asian, Caribbean and American, and Hispanic populations, but to date these have not been linked to in vivo alterations in metformin efficacy [26, 28, 39–48]. However, the impact of *SLC22A3* variants conferring increased activity is less well understood and this is the first study to assess this in vivo.

Our findings have potential implications for the use of metformin for treating diseases such as diabetes. Overall, the data suggest that human carriers of the OCT3 44M variant may show an initially strong glucose lowering response upon initiation of metformin that diminishes over time. While the loss of efficacy after long-term treatment with metformin in the OCT3 44M mice may not be beneficial for metabolic effects of metformin, it could conversely be better for tolerance to the drug. This is because GDF-15 is known to be involved in the gastrointestinal intolerance [49, 50], and metformin is also known to induce gastric disturbance in some patients which can limit compliance [51, 52]. Thus, our findings showing a lower metformin-induced increase in GDF-15 levels after 14 days of metformin treatment could mean metformin is better tolerated in carriers of the OCT3 44M variant, albeit with potential reduction in efficacy.

Our findings also reinforce the importance of ethnic-specific approaches in developing precision medicine strategies for treating metabolic diseases [2, 23]. Our data demonstrate that, while the rs8187715 variant in *SLC22A3* is very rare in other parts of the world, it is present at a minor allele frequency of over 15% in Māori and Pacific peoples and so any impacts of the variant will have important implications specifically for this population. Thus, the variant could potentially act as a meaningful ancestral-specific genetic biomarker to guide precision medicine strategies of not only metformin treatment in type 2 diabetes, but also other drugs that are known to be transported by OCT3. Based on the population size, we estimate there are more than 200,000 carriers of the variant in Aotearoa NZ. Given the incidence of type 2 diabetes in Polynesian peoples, this means there are around 20,000 people with this variant who are using metformin in Aotearoa NZ [53].

Finally, it remains unknown whether the same effects on metformin uptake and efficacy occur in human carriers of the variant, so future studies will be required to resolve this.

## Supplementary information

Below is the link to the electronic supplementary material.

ESM Figs (PDF 279 KB)

## Data Availability

The datasets generated from the human cohort are not publicly available due to ethical approval requirement, but are available on reasonable request. Preclinical datasets generated from this study are available from the corresponding author on reasonable request. Any requests should also include evidence they have addressed the guidelines for working with Māori in medical genomics: https://www.waikato.ac.nz/assets/Uploads/Research/Research-institutes-centres-and-groups/Centres/MIGC/Te-Mata-Ira-Genome-Research-Guidelines.pdf. All software used in the analyses were open source and are described in the Methods.

## Abbreviations

GDF-15: Growth/differentiation factor 15
HFD: High-fat diet
KI: Knockin
44M: Variant with methionine at amino acid position 44
NZ: New Zealand
OCT3: Organic cation transporter 3
PMAT: Plasma membrane monoamine transporter
WT: Wild-type
