# Supplementary material for: The population-specific Thr44Met OCT3 coding variant affects metformin pharmacokinetics with subsequent effects on insulin sensitivity in C57Bl/6J mice
Source: Diabetologia. 2024 Oct 18;68(3):537–48. doi: 10.1007/s00125-024-06287-1 (PMC11832584; doi:10.1007/s00125-024-06287-1)

ESM Figures

ESM Figure 1. OCT3 (encoded by *SLC22A3*) sequence alignment and CRISPR/Cas9 guide RNA design.

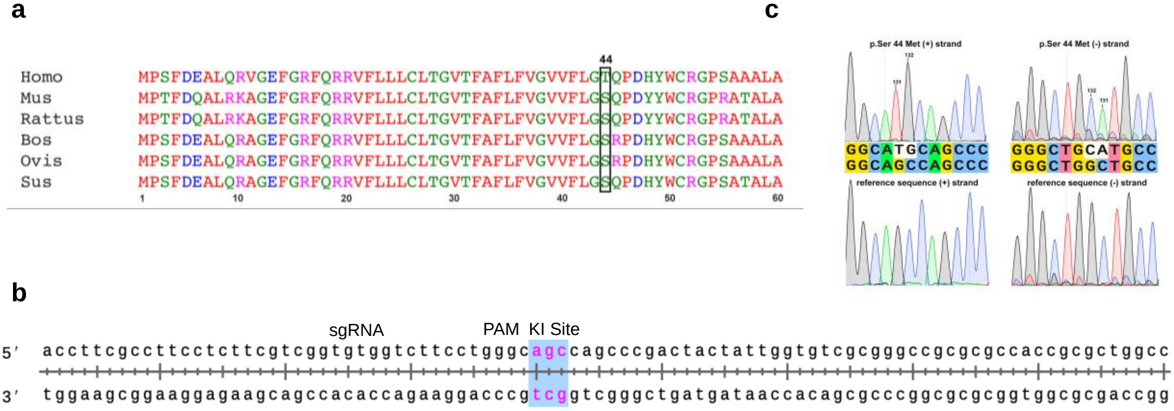

ssoDNA (127bp)

CCACTCCTCCTCGGGGCTCCAGGCGCAGCGCTCGGCCAGCGCGGTGGCGCGCGGCCCGCGACACCAATAGTAGTCGGGCTGCATGCCAAGAAAGACCACACCGACGAAGAGGAAGGC
GAAGGTGACA

ESM Figure 2. SLC22A3 variant increased metformin uptake rate after oral administration in female mice.

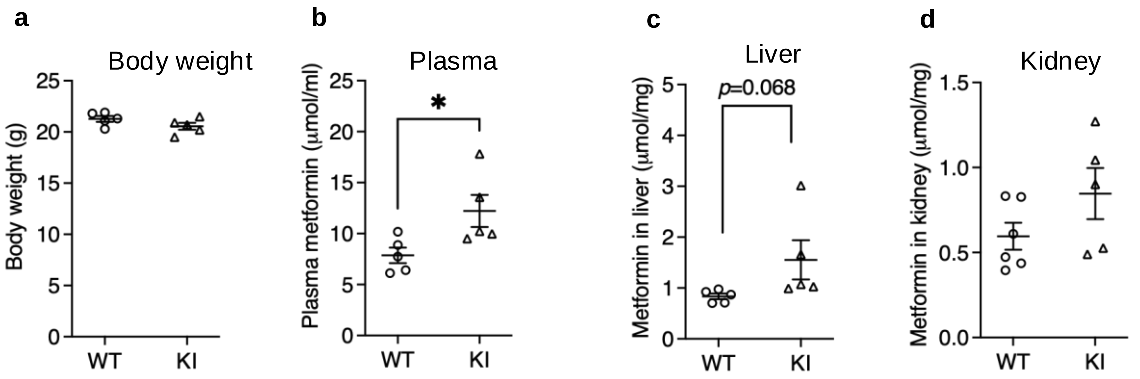

ESM Figure 3. SLC22A3 variant increased uptake rate with high dose of metformin.

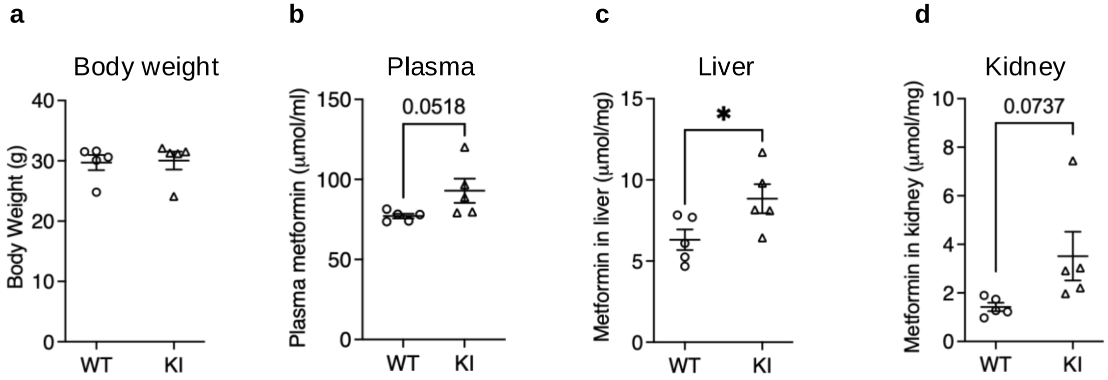

ESM Figure 4. Longer term metformin treatment has no obvious effect on plasma lactate levels.

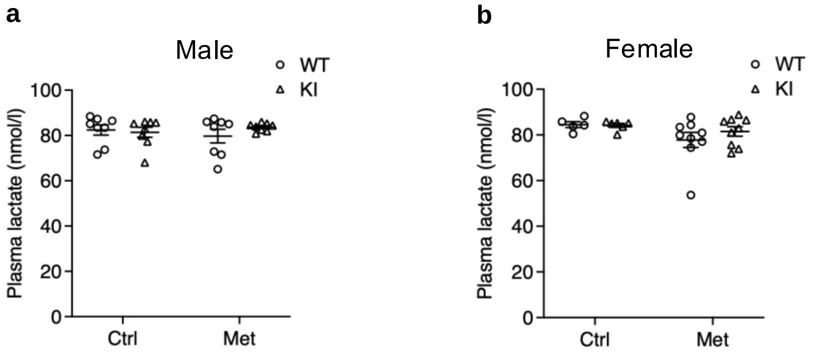

Supplement: Supplementary file 1 — ESM Figs (PDF 279 KB) [file 125_2024_6287_MOESM1_ESM.pdf]
